# A genomic signature for accurate classification and prediction of clinical outcomes in cancer patients treated with immune checkpoint blockade immunotherapy

**DOI:** 10.1038/s41598-020-77653-3

**Published:** 2020-11-25

**Authors:** Mei Lu, Kuan-Han Hank Wu, Sheri Trudeau, Margaret Jiang, Joe Zhao, Elliott Fan

**Affiliations:** 1 Department of Public Health Sciences, Henry Ford Health System, 3E One Ford Place, Detroit, MI 48202 USA; 2 Division of Data Analytics, Northern Medical Center, Middletown, NY USA; 3 Department of Computational Medicine and Bioinformatics, University of Michigan, Ann Arbor, MI USA

**Keywords:** Biomarkers, Predictive markers, Machine learning, Cancer

## Abstract

Tumor mutational burden (TMB) is associated with clinical response to immunotherapy, but application has been limited to a subset of cancer patients. We hypothesized that advanced machine-learning and proper modeling could identify mutations that classify patients most likely to derive clinical benefits. Training data: Two sets of public whole-exome sequencing (WES) data for metastatic melanoma. Validation data: One set of public non-small cell lung cancer (NSCLC) data. Least Absolute Shrinkage and Selection Operator (LASSO) machine-learning and proper modeling were used to identify a set of mutations (biomarker) with maximum predictive accuracy (measured by AUROC). Kaplan–Meier and log-rank methods were used to test prediction of overall survival. The initial model considered 2139 mutations. After pruning, 161 mutations (11%) were retained. An optimal threshold of 0.41 divided patients into high-weight (HW) or low-weight (LW) TMB groups. Classification for HW-TMB was 100% (AUROC = 1.0) on melanoma learning/testing data; HW-TMB was a prognostic marker for longer overall survival. In validation data, HW-TMB was associated with survival (p = 0.0057) and predicted 6-month clinical benefit (AUROC = 0.83) in NSCLC. In conclusion, we developed and validated a 161-mutation genomic signature with “outstanding” 100% accuracy to classify melanoma patients by likelihood of response to immunotherapy. This biomarker can be adapted for clinical practice to improve cancer treatment and care.

## Introduction

Immune checkpoint blockade immunotherapy is an emerging cancer treatment, and several regimens have been approved by the US Food and Drug Administration for treatment of melanoma and lung cancer with and without metastases, as well as renal cell carcinoma (RCC), bladder cancer, and Hodgkin’s lymphoma^1^. However, despite impressive response rates, the clinical benefit of these regimens has been limited to subgroups of patients with specific tumor types^2^ and certain interaction patterns between cancer cells and immune cells within the microenvironment^1^. There is an unmet need for biomarker identification to correctly stratify patients for proper treatment^3–5^.

Several tumor biomarkers, including Programmed Death Ligand 1 (PD-L1) immunohistochemistry and RNA gene expression profile (GEP) IFN-gamma cytolytic signatures, have been reported to predict the clinical response to immune checkpoint blockade immunotherapy, especially to anti- PD-L1 and anti-Programmed Death 1 (PD-1) immunotherapies. Nonsynonymous somatic mutations alter amino acid residues of a protein; in turn, these altered amino acid residues create new T-cell epitopes (neoepitopes) from a previously self-peptide^6,7^, serving as neoantigens capable of eliciting an antitumor immune response. Each nonsynonymous mutation increases the chances of immunogenic neoantigen formation—a probabilistic event. Therefore, tumor mutational burden (TMB)—defined as the number of nonsynonymous mutations in the tumor—can be used as a predictive biomarker for early response to immune checkpoint blockade immunotherapy.

Whole exome sequencing can be used to estimate TMB, which is directly associated with the success of checkpoint blockade immunotherapy^8–10^. For example, Snyder et al.^8^ showed that melanoma patients with a tumor mutational load ≥ 100 received significantly more clinical benefit from CTLA-4 blockade therapy than patients with mutation loads less than 100. The same researchers found that a high mutational load (> 100) was also significantly correlated with improved overall survival (OS) in learning data but failed to detect such effect on testing data. Likewise, Rizvi et al.^9^ showed that a tumor mutational load cutoff of ≥ 178 was associated with significant long-term clinical benefits in 34 patients with non-small cell lung cancer (NSCLC) treated with PD-1 blockade therapy compared to patients with lower mutation loads; they also observed that a high mutational load (> 178) was significantly associated with improved progression-free survival [PFS; area under the receiver operating characteristic curve (AUROC) = 0.86]^11–16^. TMB has been found to predict clinical response to pembrolizumab monotherapy among NSCLC patients^11^ as well as in patients with a variety of rare cancers^12–15^. However, TMB did not predict clinical response to pembrolizumab combined with chemotherapy or nivolumab combined with ipilimumab (the Checkmate-227 study)^17^. It is possible that TMB may play a different role in the setting of combination therapies due to differences in how combined therapies overcome tumor resistance in biomarker-defined samples^18^. Similarly, a recent study emphasized the importance of studies that explore the combination of additional biomarkers and interrogate a wider set of clinical data^16^; however, combinations of established makers that were tested using generalized linear models showed only moderate predictive ability (AUROC = 0.78). Therefore, use of TMB should be further validated in separate samples of patients treated with monotherapy and combination therapy.

Given that whole exome sequencing is costly and not regularly performed as part of routine care, the generation of mutation load markers that depend on a denominator derived from the number of gene mutations processed is not practical. As a result, several studied have attempted to evaluate TMB using a sub-sample of genes. For example, Campesato et al.^19^ used established cancer-gene panels (CGP), such as the Foundation Medicine Panel, for mutation load marker identification in NSCLC; while ability to predict six-month clinical benefit of immunotherapy was “good” (AUROC 0.72–0.84), the biomarker failed to predict overall survival. Roszik et al.^20^ used a set of 170 gene mutations to predict overall survival for patients with melanoma or NSCLC. Another recent study^21^ showed that TMB determined by the Memorial Sloan Kettering-Integrated Mutation Profiling of Actionable Cancer Targets (MSK-IMPACT) gene panel predicted survival after blockade immunotherapy across multiple cancer types in patients with advanced cancer; the cutoffs (defined as number of mutations) varied by cancer type. However, it was not clear whether those gene combinations or TMB predicted 6-month clinical benefit. Although 6-month clinical benefit of immunotherapy is a strong surrogate endpoint for overall survival, TMB biomarkers that predict clinical benefit may not predict survival, or vice versa^8–10,19,20^. There remains an unmet need for a cost efficient and more predictive biomarker to ensure that cancer patients benefit from the promise of checkpoint blockade immunotherapies.

We hypothesized that advanced machine-learning technologies and proper modeling approaches could improve biomarker predictive ability and identify a set of gene mutations that could correctly classify which patients were likely to achieve clinical benefits at six months after blockade immunotherapy initiation, and that this same set of mutations would be a prognostic marker for increased overall survival.

## Methods

We downloaded whole exome sequencing mutation data for two independent samples of melanoma patients (n = 39 and n = 25) published by Snyder et al.^8^ and a sample of NSCLC patients (n = 34) published by Rizvi^9^; links to data are available in the referenced manuscripts. For the original studies, matched normal DNA extracted from peripheral blood was used to filter germline variants and to enable unambiguous identification of somatic mutations. All patients received blockade immunotherapy [either anti-cytotoxic T lymphocyte-associated antigen (CTLA-4) or anti-PD-1]. The primary endpoint—long-term clinical benefit—was defined by radiographic evidence of freedom from disease or evidence of a stable or decreased volume of disease for more than 6 months. Patients were followed up for overall survival as the secondary endpoint.

Following the methods of Breiman et al.^22^, we used the larger melanoma dataset (n = 39) for learning data and the smaller melanoma dataset (n = 25) for testing data to develop and validate the mutation model. We then used the remaining dataset (NSCLC sample, n = 34) for external validation of the model classification ability.

### Data availability

Melanoma data is embedded in Supplementary Tables available in the Appendix to Snyder et al.^8^: https://www.nejm.org/doi/suppl/10.1056/NEJMoa1406498/suppl_file/nejmoa1406498_appendix.pdf.

NSCLC data is available in the Supplementary Materials to Rizvi et al^9^: https://science.sciencemag.org/content/suppl/2015/03/11/science.aaa1348.DC1?_ga=2.203136467.331745608.1594129794-458264062.1591795446.

### Statistical modeling for 6-month clinical benefits

**Figure 1:**
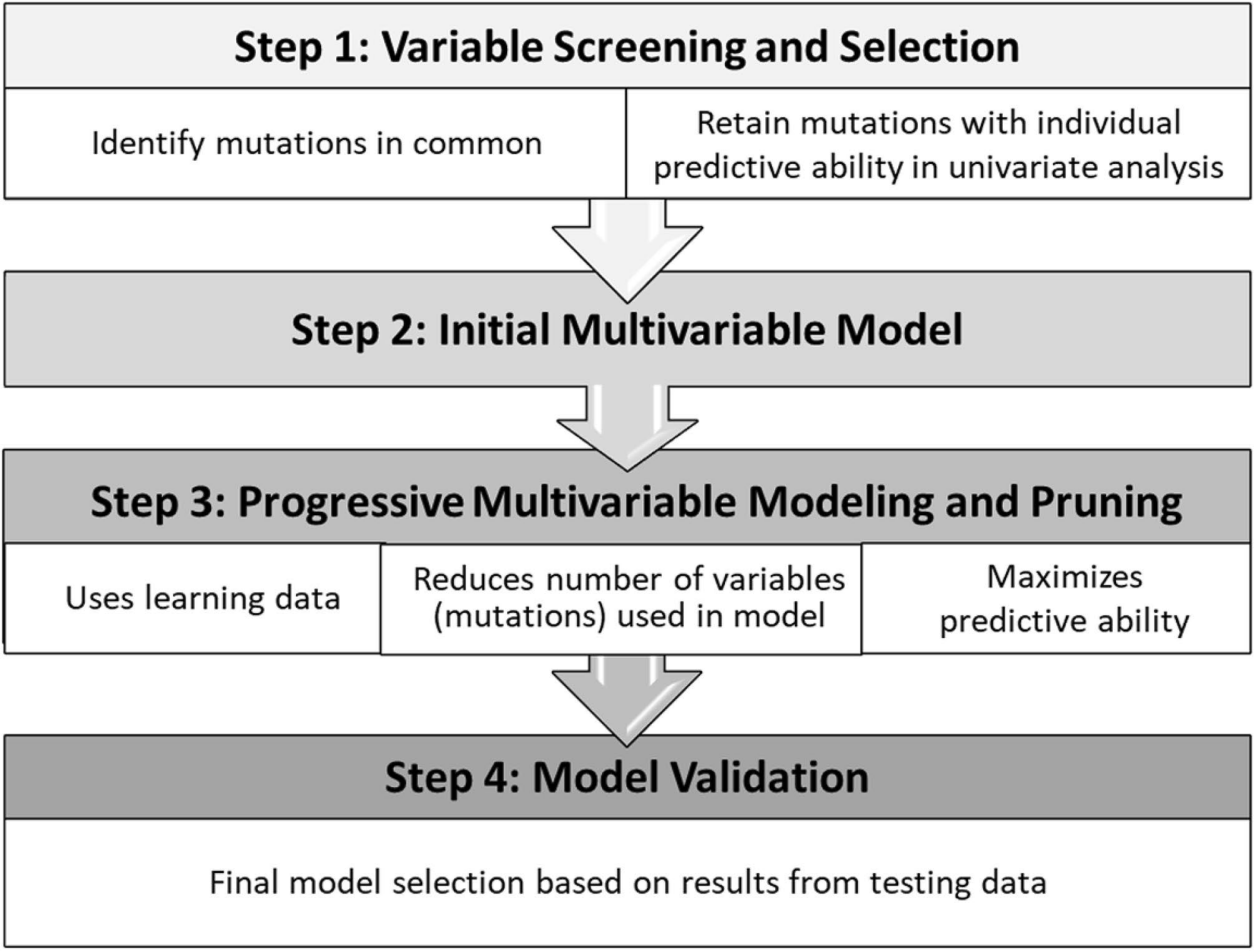
Four-step modeling process.

We used a comprehensive approach to construct our modeling process in four steps (see Fig. 1): (1) variable screening and selection; (2) creation of an initial multi-variable model by including variables with individual effects (in univariate analyses); (3) multi-variable modeling with a pruning approach to improve parsimony while retaining excellent predictive ability; and (4) model validation using independent samples.

We first selected genetic mutations common to the two Snyder datasets^8^, given the large number of mutations reported (9291–18,850) between them. To screen for mutations potentially associated with the outcomes of interest (Step 1 in Fig. 1), two-sample tests (presence and absence of long-term benefit) were performed with various cut-off p-values (0.2, 0.3, and 0.5) using learning data. Mutations were selected for inclusion in the initial multivariable model (Step 2) if they demonstrated predictive ability in the univariate models within a reasonable computational time (i.e. minutes versus hours).

A Least Absolute Shrinkage and Selection Operator (LASSO) machine-learning technique^23^ and Salford Predictive Modeler (SPM v8)^24^ was used to identify combinations of mutations with the maximum ability to predict long-term clinical benefit (Step 3 in Fig. 1). LASSO estimates the contribution of each covariate based on ordinary least squares (OLS), emphasizing combined effects rather than individual covariate effects. An elasticity option in LASSO accounts for correlations between variables and restricts the OLS estimation based on a linear combination of the sum of the absolute regression coefficients as well as on the sum of the squared regression coefficients. Pruning (Step 3) is similar to backward variable selection in a regression model; this approach was used to scale down the number of mutations/ covariates included in the model while retaining same level of the model predictive ability (as measured by AUROC) compared the first multivariable model.

The most parsimonious model that retained “excellent” goodness-of-fit (i.e., AUROC > 0.90) on the testing data was considered to be optimal. In addition, we generated a cut-off point/threshold that optimized both sensitivity and specificity to classify patients into two groups—those with a high-weight tumor mutational burden (HW-TMB) and those with a low-weight tumor mutation burden (LW-TMB). Finally, the model predictive ability (AUROC) was validated using external data.

### Testing the effect of HW-TMB on overall survival

Kaplan–Meier and log-rank tests were used to test differences in overall survival between the HW-TMB and LW-TMB groups. First, the effect of the HW-TMB marker on overall survival was tested using two independent samples from Snyder^8^, and then validated using the external NSCLC sample data^9^.

### Ethics approval and consent to participate

The study was conducted based on published data.

## Results

Patient characteristics and clinical follow-up are presented in Table 1. In general, patients’ age and sex were similar across all samples, except that patients were less likely to be male in the NSCLC sample^9^. Roughly half (41–66%) were determined to have received clinical benefit from treatment based on radiological evidence. Median follow-up was approximately 2 years in melanoma samples and 0.36 years for the NSCLC sample. The number of genetic mutations processed was 18850 in the first melanoma sample (n = 39), roughly twice that of the 9000 genes processed in the second melanoma and the NSCLC sample.

**Table 1:** Patient characteristics across study data sets.

	Snyder et al (2014)^8^	Snyder et al (2014)^8^	Rizvi et al (2015)^9^
Sample size	39	25	34
Cancer type	Melanoma	Melanoma	Lung
Metastatic (stage IV)	100%	100%	100%
Received anti-CTLA-4/PD-1	100%	100%	100%
Age, mean (+ /− STD)	59 (± 16)	61 (± 11)	62 (+ /- 8)
Male	64%	56%	47%
Long term clinical benefit	66%	44%	41%
Median follow up/total duration (in years)	2.1/7.9	2/6.9	0.36/2.3
Overall survival (OS)	49%	40%	35.3%
Total genetic mutations	18,850	9291	9049

### TMB biomarker to predict long-term clinical benefit at 6 months

We used a p-value cutoff of 0.30 to screen for genetic mutations; this yielded 2139 genes for consideration in the initial multivariable model. The first LASSO model retained 670 genetic mutations with an AUROC of 1.0 on both learning and testing data and 100% accuracy for long-term clinical benefit. Our pruning approach reduced the number of mutations in the model to 161 (Table 2); among these, only nine genes (< 6%) had negative coefficients, indicating that our weighted gene mutation combination model was consistent with previous published mutation load markers for 6-month clinical benefits of immunotherapy. One hundred percent of patients with HW-TMB demonstrated clinical benefits of immunotherapy at 6 months; classification accuracy was 100% (AUROC = 1.0 on both learning and testing data; see Table 2). In further validation using lung cancer data^9^ AUROC was 0.83.

**Table 2:** 161-gene tumor mutational burden (TMB) model: gene mutations and coefficients included in the final least absolute shrinkage and selection operator (LASSO) model.

Model for metastatic melanoma; AUROC = 1.0; prob = 1.0/(1.0 + exp(+ score)) with threshold of 0.41; ROC = 1.0 and 100% of sensitivity and specificity on testing data and 0.83 on non-small cell lung cancer
score = 0.29237 + 0.0732249 MEGF6 + 0.0741173 RNF207 + 0.0689838 TRIM63 + 0.0758446 CATSPER4 + 0.075716 DLGAP3 + 0.0750007 GBP6 + 0.0391117 NOTCH2—0.0493249 IQGAP3 + 0.0782592 QSOX1 + 0.0748039 LAMC1 + 0.0397537 DENND1B + 0.0714242 PPFIA4 + 0.0738524 KLHDC8A + 0.0402354 DYRK3 + 0.0744581 NBAS + 0.038275 PPP1R21 + 0.0392758 ZNF638 + 0.0714602 POLR1B + 0.0386346 GPR148 + 0.0386539 ZNF385B + 0.0746926 WNT6 + 0.0740007 PER2 + 0.0669716 IL17RE—0.0508015 GALNTL2 + 0.0725383 NR1D2 + 0.0395422 PRSS50 + 0.0720539 BAP1 + 0.0747932 SEMA3G + 0.0387396 GNL3 + 0.0747427 CHDH + 0.0750608 IL17RB + 0.0746505 STX19 − 0.0497445 MYH15 + 0.0387805 DZIP3 + 0.074709 B3GALNT1 + 0.0751426 YEATS2 + 0.0253181 EHHADH + 0.0391551 OTOP1 + 0.0749409 PSAPL1 + 0.0743374 NKX3_2 + 0.0790856 BEND4 + 0.0721983 CDS1 + 0.0405115 ALPK1 + 0.0754284 PET112 + 0.0748741 C4ORF45 + 0.0384309 SLC6A18—0.0504599 LIFR + 0.0754974 NEUROG1 + 0.0747565 NR3C1 + 0.0771579 NUP153 + 0.0275903 CDSN + 0.0382409 TULP1 + 0.0387858 C6ORF222 + 0.0396279 TREM2 + 0.0383106 UBR2 + 0.0383879 REV3L − 0.0338541 DSE + 0.0754582 AHI1 + 0.0385375 REPS1 + 0.0687994 HIVEP2 + 0.0382618 ACTB + 0.0391369 TRIL + 0.0747616 OGDH + 0.0751848 ABCB1 + 0.0749173 SVOPL + 0.0749532 PRSS37 + 0.0721644 ZNF775 + 0.0719478 DOCK5 + 0.0394483 PENK + 0.0368404 RUNX1T1 + 0.0385315 EIF2C2 + 0.0749093 LY6K + 0.0748032 TIGD5 + 0.0388984 ZNF707 + 0.0703273 TONSL + 0.0382672 SMARCA2 + 0.0468233 KDM4C + 0.0505405 NTRK2 + 0.0387419 C9ORF96 + 0.0739168 PPAPDC1A + 0.0400501 DOCK1 + 0.0736609 ECHS1 + 0.0398175 NUP98 + 0.0746326 ABCC8 + 0.0746822 RAG2 + 0.0743656 TMEM132A + 0.0386076 SLC22A10 + 0.0404049 CADM1 + 0.0746953 C1RL + 0.0387631 AICDA + 0.0744471 TAS2R30 + 0.0748932 PRB3 + 0.077022 OR8S1 + 0.0382468 FAM186B + 0.0749445 HSD17B6 + 0.0265496 ARHGEF25 + 0.0391377 RAB35 + 0.0384944 RXFP2 + 0.0753151 IPO4 + 0.0708389 STRN3 + 0.038761 PYGL + 0.0747415 NID2 + 0.0388318 ADAM21 + 0.0748215 ARHGAP11A + 0.0749962 SLC27A2 + 0.0409132 MRPS11 + 0.0751842 ALDH1A3 + 0.0394624 BAIAP3 + 0.0755822 E4F1 + 0.0744655 C16ORF71 − 0.0513301 SEC14L5 + 0.0753988 HS3ST4 + 0.0746807 MMP2 + 0.0123829 DNAH9 + 0.0747599 ZNF287 + 0.0391155 MPRIP + 0.0746991 ALKBH5 + 0.0751111 LLGL1 + 0.0719964 FOXN1 + 0.0754716 PIGS + 0.038593 AOC3 + 0.0492426 MYCBPAP + 0.0739014 BCAS3 + 0.0397223 INTS2 + 0.0747976 CSH1 + 0.0750126 V9_SEP + 0.0401513 BAIAP2 + 0.0754116 SLC38A10 + 0.0381561 CCDC57 + 0.0290584 ASXL3 + 0.0750522 APBA3 + 0.0396487 ZNF266 + 0.037288 MAP1S + 0.0753293 DDX49 + 0.0392096 ZNF91 + 0.0746331 PRX + 0.0407147 GRIK5 + 0.0747295 ZFP112 + 0.0755519 BCL3 + 0.0275211 ARHGAP35 + 0.0301102 GLTSCR1 + 0.0747177 ALDH16A1 + 0.074638 RPL13A + 0.0758337 VN1R2 + 0.0755278 LILRA4 + 0.0747503 STK4—0.0355315 EYA2 + 0.0746771 SCAF4 + 0.0747302 HUNK + 0.0401806 ARVCF + 0.0744056 MED15 + 0.0414217 PI4KA + 0.0391605 CABIN1 − 0.0349109 NHS + 0.0265022 WAS + 0.0751745 YIPF6 + 0.0748873 ZMYM3 + 0.0385064 TAF9B + 0.0755487 OR13H1 + 0.0394 IDS + 0.0258907 PCDH11Y

### TMB biomarker as a prognostic marker for overall survival

**Figure 2:**
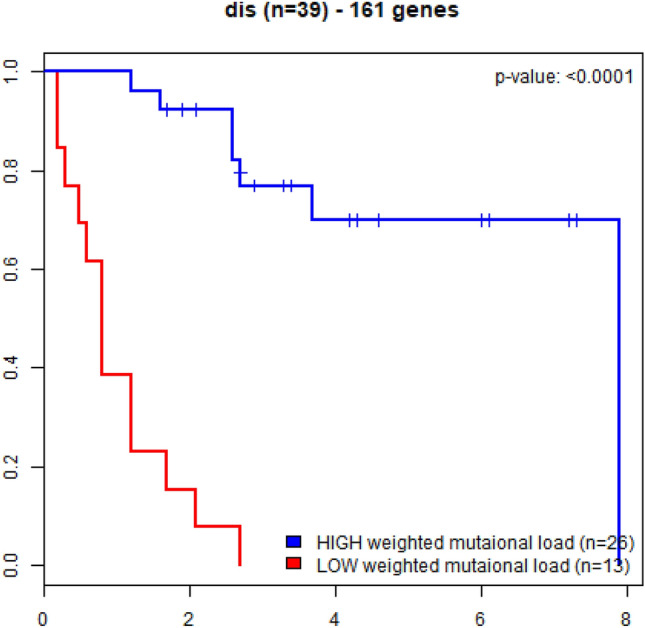
Kaplan–Meier curves for overall survival in advanced melanoma patients treated with immune checkpoint blockade immunotherapy (learning data n = 39); patients with the high-weight tumor mutation burden (HW-TMB) had significant longer survival, compared to those with low-weight tumor mutation burden (LW-TMB).

**Figure 3:**
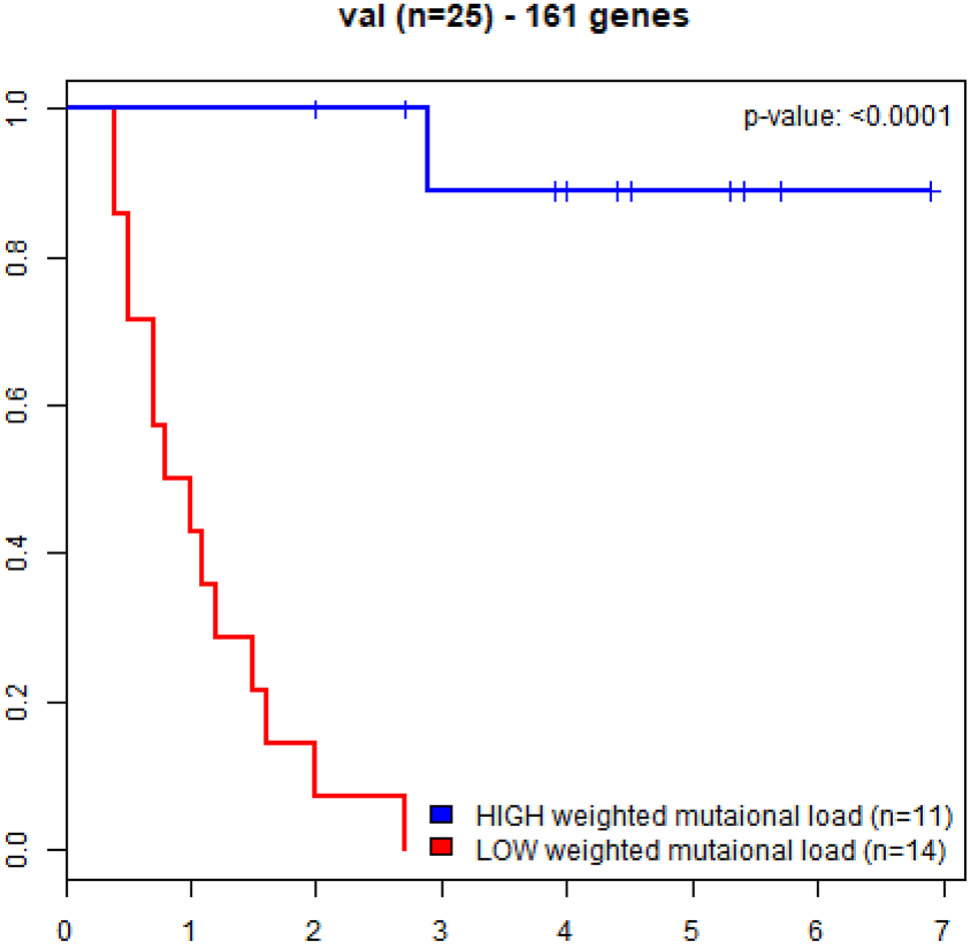
Kaplan–Meier curves for overall survival in advanced melanoma patients treated with immune checkpoint blockade immunotherapy (testing data n = 25); patients with high-weight tumor mutation burden (HW-TMB) had significant longer survival, compared to those with low-weight tumor mutation burden (LW-TMB).

**Figure 4:**
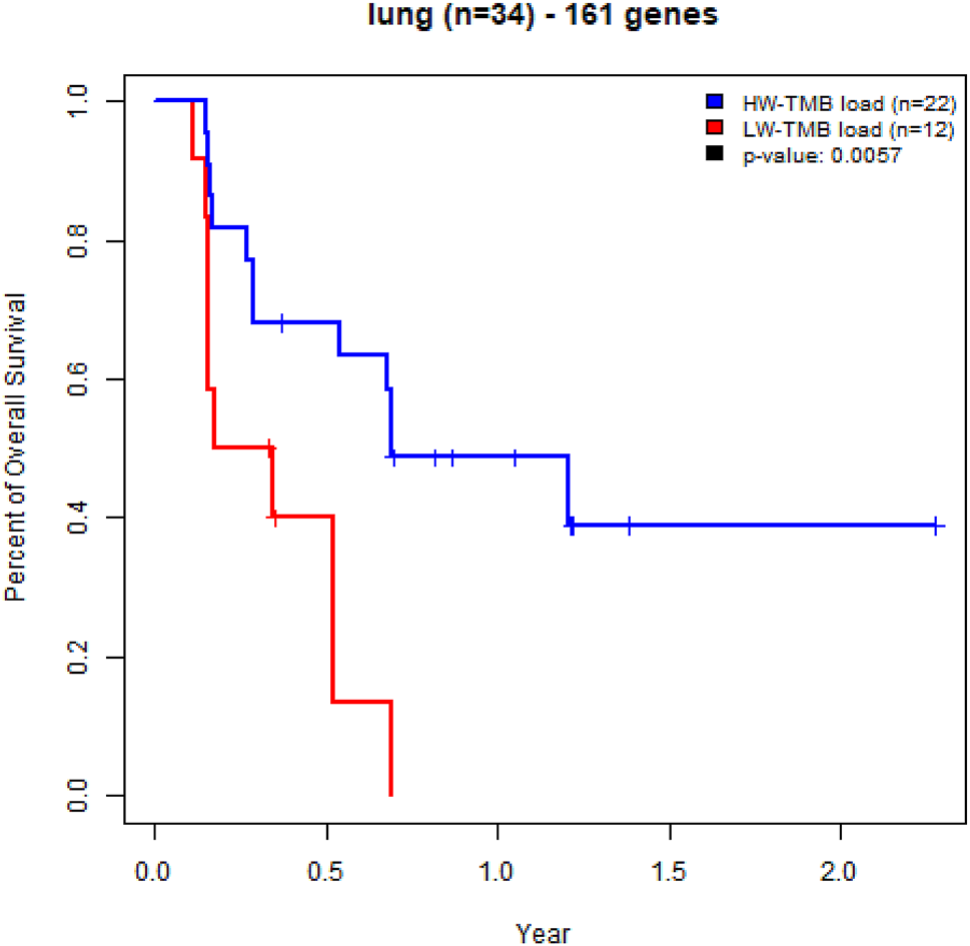
Kaplan–Meier curves for overall survival in advanced non-small cell lung cancer patients treated with immune checkpoint blockade immunotherapy (testing data n = 34); patients with high-weight tumor mutation burden (HW-TMB) had significant longer survival, compared to those with low-weight tumor mutation burden (LW-TMB).

In the melanoma learning data sample (n = 39), estimated median survival was was 7.9 years for patients with HW-TMB versus 0.8 years in patients with LW-TMB (p-value < 0.0001; Fig. 2). In the melanoma testing data sample (n = 25), estimated median survival was > 7 years in HW-TMB patients, compared to 0.9 years in LW-TMB patients (p < 0.0001; Fig. 3). In the NSCLC sample, estimated median survival was 0.69 years in HW-TMB patients versus 0.26 in LW-TMB patients (p = 0.0057; Fig. 4).

## Discussion

Our four-step proper model building process and advanced machine-learning approach resulted in a validated model based on a set of 161 mutations generated a model with an “outstanding” 100% predictive accuracy to classify melanoma patients into two groups based on tumor mutation burden. All patients in the HW-TMB group responded to immunotherapy, showing clinical benefit at six months, while all patients in the LW-TMB group were non-responders. This model also had “good” classification ability (AUROC = 0.83) among patients with NSCLC. Furthermore, melanoma patients classified as HW-TMB has median survival time approximately eightfold higher than those with LW-TMB in two independent studies (p < 0.0001). Among NSCLC patients, patients with the same HW-TMB marker had median survival almost three times that of patients in the LW-TMB group (p < 0.001).

Although previous publications have demonstrated that tumors with a high number of nonsynonymous somatic mutations are likely to respond to immune checkpoint blockade immunotherapy in patients with melanoma, these biomarkers depend on a denominator derived from the number of gene mutations processed and fail to predict a better overall survival. In addition, whole exome sequencing is not routinely available in clinical practice; the process can also be costly and time-consuming (results may take up to 5–6 weeks based on current technology). As a result, several studies have attempted to evaluate TMB using a gene panel assays with reduced turnaround time (approximately 2 weeks). Campesato et al.^19^ proposed using established cancer-gene panels (CGPs) for biomarker identification. The study showed that a mutational load cutoff point of 7 (based on a gene panel consisting of 315 genes) was comparable to a cutoff ≥ 190 points (based on whole exome sequencing) for prediction of durable clinical benefit (partial or stable response lasting > 6 months) among patients with NSCLC; a high mutational load was significantly associated with progression-free survival but no data was presented for overall survival. However, the CGP-mutational load biomarker was not associated with clinical benefit from CTLA-4 blockade therapy in melanoma patients, and there were no observed differences in overall survival between the high and low mutational load groups. Although model predictive ability (AUROC) for durable clinical benefit in NSCLC patients was moderate (0.73–0.84) based on the derivation sample, none of biomarkers predict long term overall survival. In contrast, Roszik et al.^20^ used a panel of 170 gene mutations to predict overall survival. Their study showed that a weighted 169 gene mutation combination significantly predicted overall survival for patients with melanoma or NSCLC treated with immunotherapies. However, the study was restricted to a fixed panel of gene mutations, and it was unclear whether those gene combinations predicted the clinical benefit of immunotherapy at 6 months.

To address limitations of these methods, we used a comprehensive approach to construct our modeling process in four steps (Fig. 1): (1) variable screening and selection; (2) creation of an initial multi-variable model by including variables with individual effects (in univariate analyses); (3) multi-variable modeling with a pruning approach to improve parsimony while retaining excellent predictive ability; and (4) model validation using independent samples. Our analysis focused on a subset of gene mutations to predict clinical benefits of immunotherapy at 6 months among patients with metastatic melanoma. As described above, whole exome sequencing usually identifies a large quantity of mutations which may vary across studies; this creates challenges for model development and validation. We found that the process of screening potential mutations of interest was an essential first step for a successful model-building process. In the current analysis, we used p-values ranging from 0.1 to 0.5 for individual genetic mutation selection before multivariable modeling; in this analysis, a p-value of 0.3 was chosen in order to retain a sufficient number of gene mutations for the initial multivariable model, given that lower p-values did not identify enough mutations to be useful for the modeling analysis, and higher p-values required too much computational time and added noise to the model.

Our advanced machine learning LASSO method, which emphasizes the combined effects of multiple mutations, generated a model that predicted both long-term clinical outcomes and overall survival across two cancer types among patients who received blockade immunotherapies. Because overall survival is a commonly used outcome for testing treatment efficacy in cancer research and clinical practice, our marker may be useful for identifying appropriate patients for better treatments and care.

We also note that the final HW-TMB model was not unique. The first model retained as many as 670 gene-mutation combination and yielded AUROC = 100% predictive accuracy based on the testing data. During model pruning process, we trimmed the number of mutations until the model reached 161 mutations, the smallest subset as the final model which continued yielding AUROC = 100% on testing data. This 161-mutation HW-TMB biomarker has much better predictive ability for both clinical benefit at 6 months and overall survival than previously published biomarkers based on either whole exome sequencing data or subsets of whole exome sequencing^8,9^.

Recently, Samstein et al.^21^ showed that TMB, defined as mutation load based on a pre-defined subset of 468 cancer-related genes from MSK-IMPACT, predicted survival after blockade immunotherapy across multiple cancer types in patients with advanced cancer. However, TMB cutoffs derived from targeted sequencing (gene panel)s may vary across cancer types. Our results are consistent with the Samstein results^21^, despite the difference in TMB measures as well as the study populations. Our 161 gene-mutation signature/marker was a prognostic marker for survival in patients with one of two advanced stage cancers (melanoma or NSCLC) treated with blockade immunotherapy. This supports the use of 6-month clinical response as a surrogate endpoint for overall survival and the use of a 6-month clinical response biomarker as a surrogate for survival. Another study^16^ has interrogated established markers and tested their predictive ability for a wider set of clinical endpoints; result were only moderately predictive (AUROC = 0.78). One limitation of that study could be the inclusion of both monotherapy and combination therapy data; the role of TMB in monotherapy should be studied separately from combination therapy due to different resistance mechanisms overcome by combined agents.

As shown in Table 2, our marker accounts for both the weight and value of mutations, which allows us to reduce the number of genes included in the model. This parsimony translates into significantly reduced computational processing time, compared to whole exome sequencing, without loss of predictive accuracy—resulting in a marker that can be adapted for clinical practice, allowing patients to receive appropriate treatment in a timely fashion with clear expectations regarding the clinical benefits.

There are a number of limitations to our analysis. First, the 161 gene-mutation signature is hypothesis-driven; however, the relationship of TMB—measured by either a set of mutation genes or a total mutation cut off—to clinical response in immune monotherapy is well recognized. It is possible that the gene mutations identified in our model may be associated with well-studied genes or may be new candidates for study. A goal for future work includes investigation of the correlation between these new and previously recognized genes to determine their biological relevance. Second, although we have used only a subset of mutations in our analysis, implementation of this method in clinical practice may still be challenging, given the number of genes included. However, compared to whole-exome sequencing or CGPs based on 315 genes, our method may be more cost-efficient. Although our analysis was focused on development and validation among patients with melanoma, we observed “good” predictive ability (AUROC = 0.83) for NSCLC outcomes in our external validation. It is likely that a more specific biomarker would improve predictive ability for patients with NSCLC, but such an exercise was outside the scope of the current analysis, given the small dataset and a lack of existing external data for validation. Finally, while our biomarker was validated using external melanoma data, the validation dataset was derived from patients from the same institution or using the same whole exome sequencing processing technology as the learning data. We were unable to validate our model using published metastatic melanoma data from Hugo et al.^25^ (n = 37) or by Van Allen et al.^26^ (n = 110) because there were differences in the whole exome sequencing technology used. Future work will include validation of our marker using a larger independent external dataset based on the same whole exome sequencing technology, evaluated against the same outcomes of interest.

## Conclusions

Although previous studies showed that TMB (defined as the number of mutations) predicted the six-month clinical benefits of blockade immunotherapy, predictive ability of these biomarkers was only “good;” these markers also failed to accurately predict overall survival. Our four-step proper model building process and advanced machine-learning approach generated a 161-HW-TMB that predicted six-month clinical response to treatment with 100% discrimination ability (AUROC = 1.0). This same set of mutations, further validated using external data sets, was a prognostic marker for overall survival among patients with melanoma and lung cancer, regardless of heterogeneous patient characteristics, previous treatment status, or type of immunotherapy. We believe our biomarker shows great promise to be adapted for clinical practice and will improve cancer treatment and care.

## Data Availability

All data generated or analyzed during this study are included in this published article.

## Abbreviations

RCC: Renal cell carcinoma
TMB: Tumor mutational burden
OS: Overall survival
NSCLC: Non-small cell lung cancer
PFS: Progression-free survival
AUROC: Area under the receiver operating characteristic curve
MSK-IMPACT: Memorial Sloan Kettering-Integrated Mutation Profiling of Actionable Cancer Targets
CGP: Cancer-gene panels
CTLA-4: Anti-cytotoxic T lymphocyte-associated antigen
PD-1: Anti-programmed cell death protein
LASSO: Least absolute shrinkage and selection operator
OLS: Ordinary least squares
HW-TMB: High-weight tumor mutational burden (HW-TMB)
LW-TMB: Low-weight tumor mutation burden (LW-TMB)
